# Galanin Receptors (GalR1, GalR2, and GalR3) Expression in Colorectal Cancer Tissue and Correlations to the Overall Survival and Poor Prognosis of CRC Patients

**DOI:** 10.3390/ijms23073735

**Published:** 2022-03-29

**Authors:** Jacek Kiezun, Janusz Godlewski, Bartlomiej E. Krazinski, Zygmunt Kozielec, Zbigniew Kmiec

**Affiliations:** 1 Department of Human Histology and Embryology, Faculty of Medical Sciences, University of Warmia and Mazury in Olsztyn, 10-082 Olsztyn, Poland; janusz.godlewski@uwm.edu.pl (J.G.); bartlomiej.krazinski@uwm.edu.pl (B.E.K.); 2 Department of Pathomorphology, Faculty of Medical Sciences, University of Warmia and Mazury in Olsztyn, 10-561 Olsztyn, Poland; zygmunt.kozielec@uwm.edu.pl; 3 Department of Histology, Faculty of Medicine, Medical University of Gdansk, 80-211 Gdansk, Poland; zkmiec@gumed.edu.pl

**Keywords:** colorectal cancer, galanin receptors, immunohistochemistry, overall survival

## Abstract

Colorectal cancer (CRC) is the second most common cause of cancer in women and the third in men. The postoperative pathomorphological evaluation of patients with CRC is extremely important for future therapeutic decisions. Although our previous studies demonstrated high galanin (GAL) presence within tumor tissue and an elevated concentration of GAL in the serum of CRC patients, to date, there is a lack of data regarding GAL receptor (GalR) protein expression in CRC cells. Therefore, the aim of this study was to evaluate the presence of all three types of GalRs (GalR1, GalR2 and GalR3) within epithelial cells of the human colon and CRC tissue with the use of the immunohistochemical method and to correlate the results with the clinical-pathological data. We found stronger immunoreactivity of GalR1 and GalR3 in CRC cells compared to epithelial cells of the unchanged mucosa of the large intestine. No differences in the GalR2 protein immunoreactivity between the studied tissues were noted. We also found that the increased immunoexpression of the GalR3 in CRC tissue correlated with the better prognosis and longer survival (*p* < 0.0079) of CRC patients (*n* = 55). The obtained results suggest that GalR3 may play the role of a prognostic factor for CRC patients. Based on data from the TCGA-COAD project deposited in the GDC Data Portal, we also found that *GalR* mRNA in cancer samples and the adjacent normal tissue did not correlate with immunoexpression of the GalR proteins in CRC cells and epithelial cells of the unchanged mucosa.

## 1. Introduction

The postoperative pathomorphological evaluation of patients with colorectal cancer (CRC) is extremely important for future therapeutic decisions, especially in individuals with the regional stage of the neoplasm. These decisions include the implementation of adjuvant chemotherapy or a schedule of appropriate follow-up. Traditionally, this pathomorphological assessment is based on the TNM staging system where the cancer features, such as tumor size (T), invasion of regional lymph nodes (N) and distant metastases (M), play an essential role [1,2].

The development of the molecular targeted chemotherapy has caused a need to extend the CRC postoperative evaluation by assessing the presence of biologically active molecules and their cellular receptors within the cancer tissue. Our previous studies concerning changes of galanin expression in the vicinity of CRC invasion demonstrated high galanin (GAL) presence within tumor tissue and an elevated concentration of GAL in serum of CRC patients [3]. 

GAL is a neuropeptide that, in humans, consists of 30-amino acids and is widely distributed in the central nervous system, endocrine system, and enteric nervous system components of the gastrointestinal tract. This protein modulates intestinal motility via direct action (smooth muscle contraction) or by modulating the activity of other neuropeptides, which finally reduces intestinal motility [4]. GAL interacts with three specific receptors: galanin receptor 1 (GalR1), galanin receptor 2 (GalR2), and galanin receptor 3 (GalR3), which all belong to the family of G-protein-coupled receptors (GPCRs). 

These receptors trigger two major signal transduction pathways: GalR1 and GalR3 stimulate cAMP synthesis, and GalR2 stimulates phosphatidylinositol (IP_3_) synthesis [5]. GalRs are involved in a range of physiological and pathological processes including the development of neurons and neuronal regeneration and protection, blood pressure regulation, nociception, food intake, mood, water balance, energy expenditure and reproduction [6,7,8,9,10,11]. GalRs play a role in the regulation of depression-like behavior. GalR1 and GalR3 may mediate the pro-depressive and GalR2 anti-depressive effects of galanin [6,7]. 

The antinociception was induced by GalR1 via the protein kinase A (PKA) signaling pathway in rats with neuropathic pain [8]. A high level of GalR2 protects mouse hippocampal neurons against amyloid toxicity [9]. The loss of GalR3 results in higher local and systemic inflammatory cytokine levels in an experimental murine colitis model [10]. The presence of GalRs within the human gastrointestinal tract was investigated in stomach epithelial cells and gastric cancer cells [12]. Thus far, only GalR1 mRNA was detected in human colon epithelial cells and colon-derived cell lines [13,14] and also in CRC cells [15]. 

There is no data concerning the expression of GalRs in CRC cells compared with the normal intestinal epithelium of the human large intestine. Therefore, the aim of our study was to determine the presence of all three types of GAL receptors within epithelial cells of the human colon and CRC tissue by the use of the immunohistochemical (IHC) method and correlate it with the clinical-pathological data. The evaluation of GalR immunoreactivity within CRC tissue could shed more light on the role of GAL in the pathogenesis of colorectal cancer [16].

## 2. Results

### 2.1. The GalR1 and GalR3 Immunoreactivities Are Stronger in Cancer Cells Compared to Epithelial Cells of the Unchanged Mucosa of the Large Intestine

**Figure 1:**
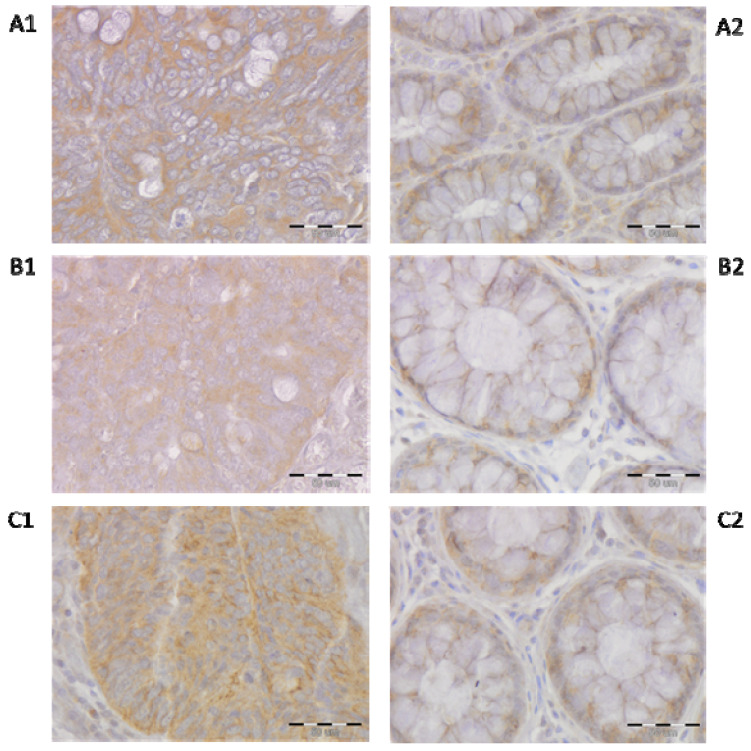
Immunohistochemical expression of galanin receptors (GalR1, GalR2 and GalR3) in colorectal cancer cells ((**A1**,**B1**,**C1**), respectively) was compared with their immunoreactivity in the epithelial and stromal cells of the unchanged mucosa of the same representative colorectal cancer (CRC) patients ((**A2**,**B2**,**C2**), respectively), as in Figure 1. The data shown here are representative for 10 slides of CRC tissue and unchanged mucosa tissue. Total magnification: 400×, the size of the scale bar in the figure is 50 µm for each microphotograph.

The immunoexpression of GalR1 and GalR3 proteins was confined to the cell membrane and cytoplasm of CRC cells and epithelial cells (enterocytes and goblet cells) of the unchanged mucosa of the large intestine (Figure 1) with various intensities of the immunoreactivity. In addition, the immunoreactivity of GalR1-3 proteins was observed in the cell membrane and cytoplasm of intestinal stromal cells, the myenteric plexuses and smooth muscle cells (unpublished). 

**Figure 2:**
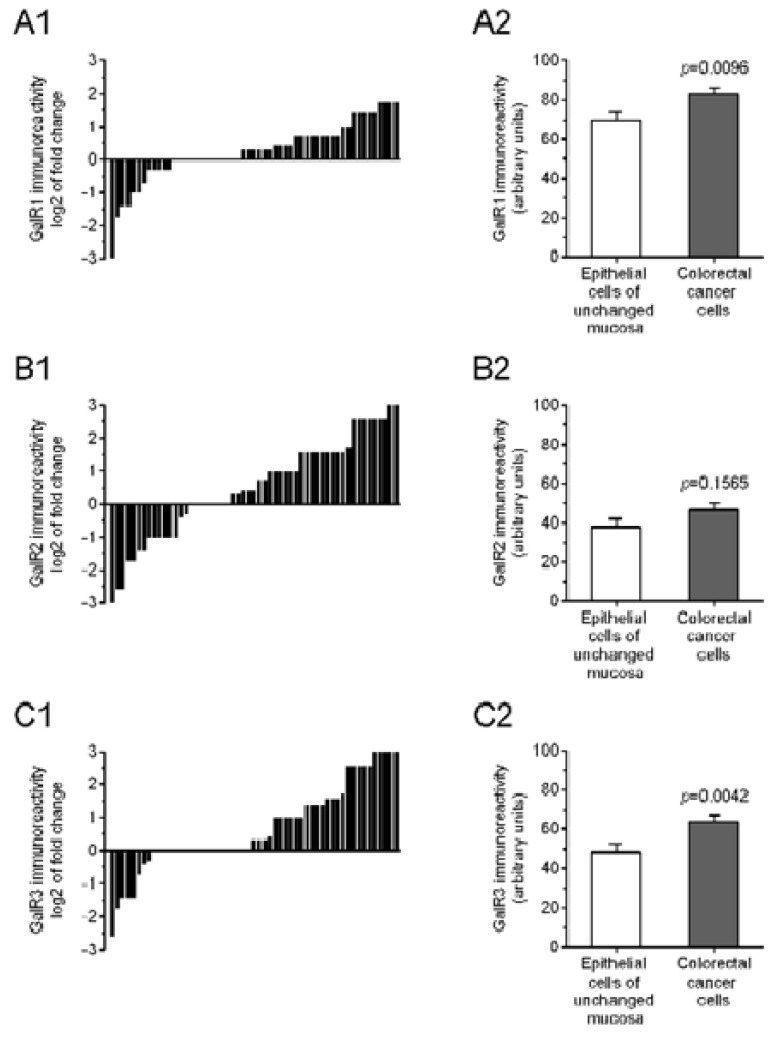
Immunoreactivity of three types of galanin receptor GalR1 (**A1**), GalR2 (**B1**) and GalR3 (**C1**) proteins in tumor sections of individual colorectal cancer (CRC) patients is shown in relation to their immunoreactivity in matched sections of unchanged mucosa. The average immunoreactivity of GalR1 (**A2**), GalR2 (**B2**), and GalR3 (**C2**) proteins in CRC cells (grey bars) compared with their immunoexpression in the epithelial cells of unchanged mucosa (white bars) was determined as described in the Methods (*n* = 55).

As shown in Figure 2 the average GalR1 immunoreactivity was significantly higher in CRC cells compared with epithelial cells of the unchanged mucosa (82.91 ± 3.16 and 70.00 ± 3.42, respectively, *p* = 0.0096). Similarly, the GalR3 immunoreactivity of tumor cells was significantly higher than that of epithelial cells of the unchanged mucosa (63.64 ± 3.28 and 48.36 ± 4.32, respectively, *p* = 0.0042). However, the average GalR2 immunoreactivity of the CRC cells did not differ from that in epithelial cells of the unchanged mucosa (46.55 ± 3.68 vs. 38.00 ± 3.98, respectively, *p* = 0.1546).

### 2.2. Fluorescent Immunolocalization and Protein Content of GalRs in Colorectal Cancer Cells and Wall of the Unchanged Large Intestine

**Figure 3:**
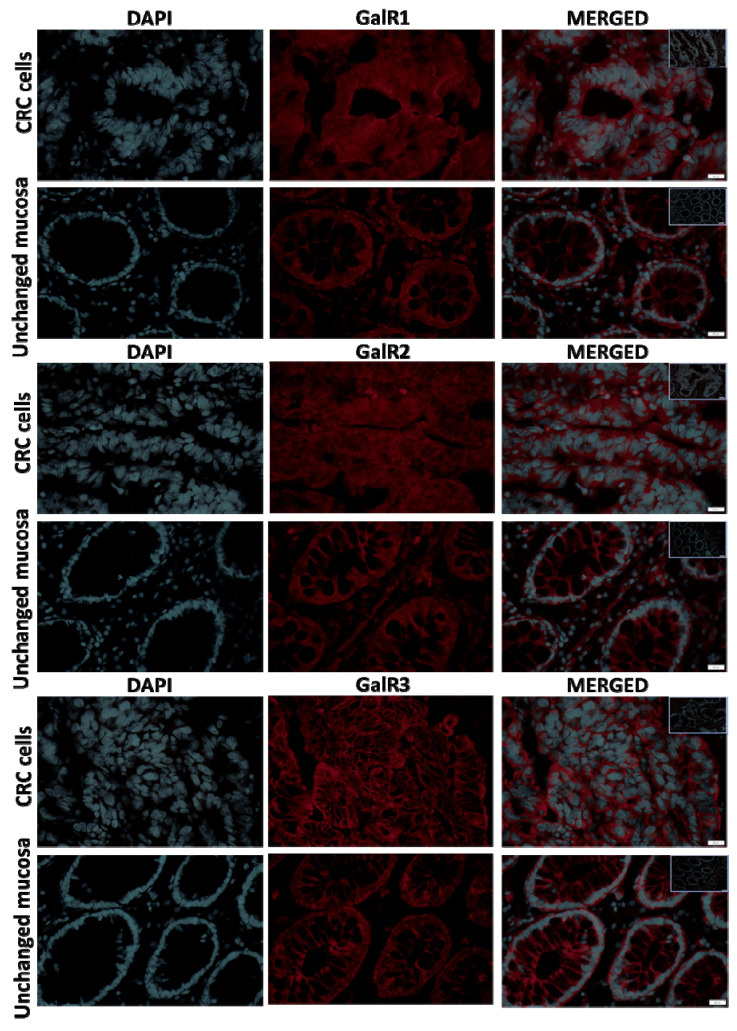
The immunolocalization of GalR1, GalR2 and GalR3 proteins was confined to the cell membrane and cytoplasm of colorectal cancer cells and epithelial cells (enterocytes and goblet cells) of the unchanged mucosa of the large intestine (Figure 3). In addition, the immunoreactivity of GalR1-3 proteins was observed in the cell membrane and cytoplasm of intestinal immune/stromal cells, the myenteric plexuses and smooth muscle cells (unpublished). Negative control was added as inserts in each merged microphotograph.

The immunoexpression of GalRs proteins was confined to the cell membrane and cytoplasm of CRC cells and epithelial cells (enterocytes and goblet cells) of the unchanged mucosa of the large intestine (Figure 3). 

**Figure 4:**
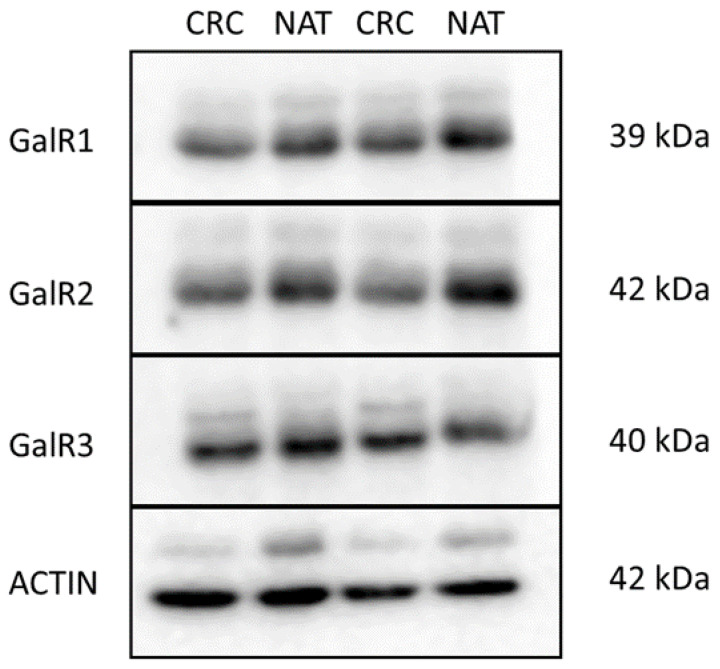
GalR1-3 proteins levels in the colorectal cancer tissue (CRC) and normal adjacent tissue (NAT) of large-intestine tissues of colorectal cancer patients (*n* = 5). Representative blots of studied tissues are shown above the graph. β-actin (ACTB) was used as the loading control.

In order to verify the specificity of the primary antibodies, we have also performed the Western Blot analyses of the GalRs proteins accumulations in CRC tissue and in the wall of the unchanged large intestine (Figure 4).

### 2.3. Low Immunoexpression of GalR3 Protein Is Correlated with Poor Prognosis of CRC Patients

**Figure 5:**
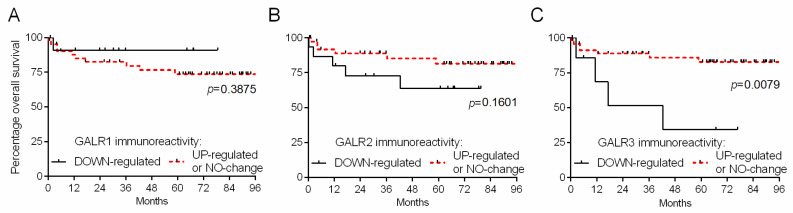
Kaplan–Meier diagrams that show the relative immunoexpression of GalRs regarding the overall survival of colorectal cancer (CRC) patients (*n* = 55) (**A**–**C**).

We divided CRC patients based on the relative immunoreactivity of GalR specimens into two groups regarded as ‘down-regulated’ (relative GalR immunoreactivity < 1) and ‘no change or up-regulated’ (relative GalR immunoreactivity ≥ 1) to obtain survival curves according to the Kaplan–Meier method. We found that GalR1 and GAlR2 relative immunoreactivity (comparing CRC cells vs. normal epithelial cells) did not correlate with the overall survival and clinical-pathological data of CRC patients (Figure 5A and Figure 5B, respectively).

However, Kaplan–Meier plots demonstrated that lower relative (CRC cells vs. normal epithelial cells) GalR3 immunoreactivity presence was associated with shorter survival of CRC patients (Figure 5C).

**Figure 6:**
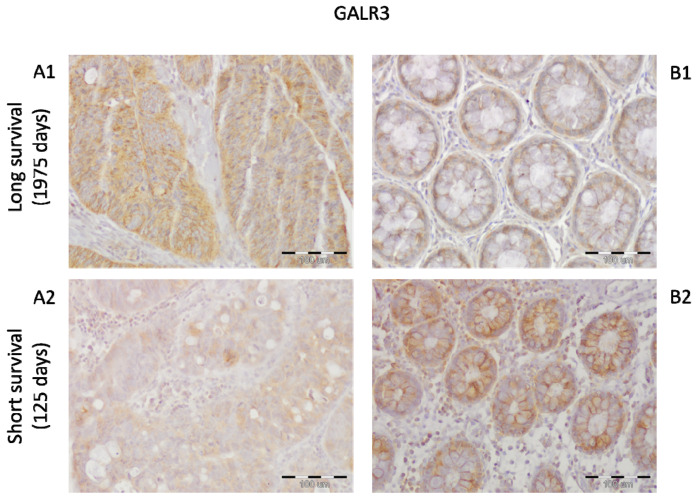
Immunoexpression of the GalR3 protein in colorectal cancer tissue (**A**) and epithelial cells of unchanged mucosa (**B**) of a colorectal cancer (CRC) patient with long survival and better prognosis ((**A1**,**B1**), respectively) and a CRC patient with shorter survival and poorer prognosis ((**A2**,**B2**), respectively). The data shown here are representative of 10 slides of CRC and unchanged tissue. Total magnification: 200×, the size of the scale bar in the figure is 100 µm for each microphotograph.

Colorectal cancer patients (*n* = 8) with shorter survival and poorer prognosis exerted a lower immunoreactivity of GalR3 compared to higher expression of this protein in non-cancerous epithelial cells (Figure 6A2,B2). An inverse relationship was found in cancer tissue of CRC patients (*n* = 46) with a better prognosis and longer survival (Figure 6A1,B1). 

Moreover, the lower relative GalR3 distribution is correlated with the higher cancer clinical stage (size of the primary tumor—T, spread to regional lymph nodes—N, Table 1).

Univariate Cox proportional hazards regression revealed that lower relative GalR3 protein immunoexpression, as well as the presence of distant metastases and lymph node metastasis, were associated with the overall survival of the patients (Table 2). Although univariate Cox regression shows that lower expression of GalR3 is connected with poor prognosis (*p* = 0.0082), a multivariate Cox regression is close to considering GalR3 (*p* = 0.0663, Table 2) as an independent prognostic factor. The subsequent multivariate analysis confirmed that only distant metastases (HR = 56.50; *p* = 0.0006, Table 2) achieved a status of an independent prognostic factor of the overall survival of CRC patients.

### 2.4. The GalR Gene Expression in Human Colorectal Cancer Database and Normal Adjacent Tissue Are Not Correlated with the Immunoexpression of the GalR Proteins

**Figure 7:**
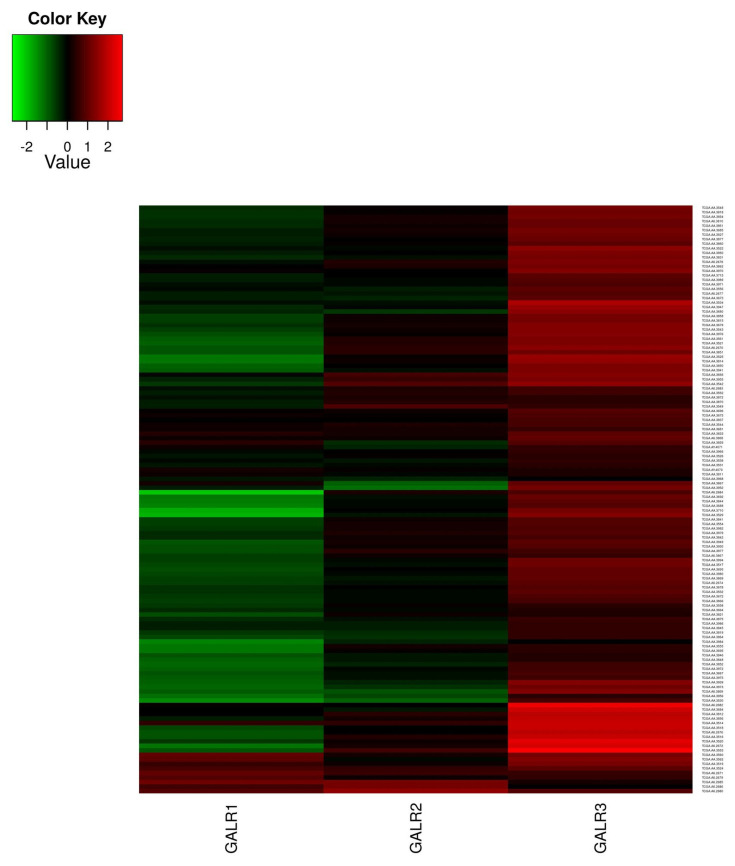
The normalized expression values of *GalR* mRNA from the TCGA COAD database. The columns describe *GalR* mRNA members, the rows show the ID of patients participating in colorectal cancer research. The bottom seven rows describe the expression of *GalR* mRNA in an adjacent normal part of the colon (*n* = 7). The remaining rows (*n* = 129) indicate the expression of *GalR* mRNA within colorectal cancer.

Data collected from the database (TCGA-COAD project deposited in GDC Data Portal, https://portal.gdc.cancer.gov/, accessed on 15 November 2021) revealed that the GalR1 and GalR2 mRNAs levels were higher in normal adjacent tissue; however, the GalR3 gene expression was stable in both cancer and adjacent normal tissue (Figure 7). We found no correlation between the GalR mRNA levels in cancer samples according to the TCGA-COAD database, the adjacent normal tissue, and the measured immunoreactivity of the GalR proteins in colorectal cancer cells as compared to the epithelial cells of the unchanged mucosa.

## 3. Discussion and Conclusions

The neuropeptide galanin may play a role in cancer progression, however, the available evidence suggests that the effects may be cancer type—dependent. Our group was the first to report increased serum galanin levels in 68 CRC patients in comparison to healthy subjects and increased concentration of galanin protein in the homogenates of CRC tumor tissue and non-involved mucosa as compared with paired samples of the tumor-adjacent mucosa [3]. 

Moreover, Kim et al. reported increased galanin mRNA levels in the tumor tissue of 116 CRC patients and the colon cancer cells of lines LOVO, HCT15, SW480, and SW620 [17]. Since in the gastrointestinal tract galanin may act on three types of its receptors [18], we decided to explore the immunoreactivity of galanin receptors in CRC tumor tissue and paired unchanged mucosa. To the best of our knowledge, this study, for the first time, demonstrates the presence of GalR1, -2, and -3 proteins in the colorectal cancer tissue and the epithelium of unchanged large intestine and reveals that GalR1 and GalR3 immunoreactivities are stronger in CRC cells compared to epithelial cells of the unchanged mucosa.

The other important finding of our study is the demonstration of the association between the low immunoreactivity of the GalR3 but not type 1 and type 2 galanin receptors and the short overall survival of the CRC patients. 

The data on the cellular distribution of GalR3 in epithelial cells of the large intestine are sparse. The association between lower expression of GalR3 in the CRC tumor tissue and worse patient prognosis noted in this study may be associated with the anti-proliferative action of GAL, which was demonstrated by a few authors in head and neck squamous cell carcinomas [19], gastric cancer cells [20] and glioma cells [21]. It has been shown in humans and experimental studies that the activation of GalRs initiates the PI3K/Akt-dependent pathway, which results in the modulation of cell proliferation [18]. 

Sugimoto et al. identified galanin and GalR1 as candidate genes suppressors of oncogenesis in ten squamous cell carcinoma cell lines (SCC) and documented increased mRNA levels of galanin and GalR1 and GalR3 in 26 clinical samples of head and neck SCC (HNSCC) tumors [22]. The anti-proliferative activity of galanin was demonstrated in the HNSCC UM-SCC-23 cell line [19]. Moreover, galanin significantly suppressed the proliferation of U251 and T98G glioma cells via GalR1 signaling as well as tumor growth in nude mice [21]. 

It has been suggested that GalR3 expression was significantly associated with high-grade glioma [23]. The overexpression of galanin in gastric cancer cells with suppressed expression of *galanin* gene resulted in increased cell apoptosis mediated by GalR3 [20]. Corresponding to these observations, the lower immunoexpression of GalR3 in the CRC patients with worse prognosis noted in this study may be associated with anti-tumor action of GAL, which is mediated by GalR3. Among GAL receptors that can be detected by immunohistochemistry, GalR3 immunoreactivity appears to be the most important prognostic factor for CRC patients.

Interestingly, in contrast to the immunoexpression of GalR3, we found a clear discrepancy between the high immunoreactivity of GalR1 in the tumor tissue of CRC patients and the lack of its association with overall survival. These findings may suggest a different role of galanin type 1 and type 3 receptors in the progression of colorectal cancer. Further studies in vitro on various CRC cell lines are necessary to explain the mechanisms of the GalR1 and GalR3 expression on CRC progression since they share similar signaling pathways. 

It has been shown that GalR1 and GalR3 predominantly couple to Gi/o subunit of G-protein what decreases intracellular cyclic AMP level, inactivates protein kinase A (PKA) and, finally, down-regulates the phosphorylation of cyclic AMP-responsive element-binding factor [23]. In this context, it is worth mentioning the bioinformatics/experimental study by Stevenson et al. who reported up-regulated levels of galanin mRNA in biopsies of hepatic metastases in eight CRC patients [15]. 

They showed that in vitro galanin or GalR1 silencing in HCT116 CRC cell line increased apoptosis and the sensitivity of the cells to 5-fluorouracil or oxaliplatin treatment. Moreover, on the basis of a publicly available CRC data set, they attributed high GalR1 mRNA levels to the better prognosis of CRC patients with early stages of the disease [15]. However, it has to be noted that the up-regulated mRNA levels do not always correspond to the increased protein expression, and, therefore, the results of our present immunohistochemical study, which suggest the importance of the GalR3 immunoexpression as a predictive factor for CRC progression seem to be more relevant for clinical medicine.

In contrast to CRC, the expression of galanin and its receptors in other cancers of the gastrointestinal tract was found to be decreased, unchanged or decreased. For instance, Zhang L et al. showed that galanin plasma levels and tumor galanin mRNA levels were significantly decreased in patients with gastric cancer [12].

In our studies, significantly higher GalR1 immunoreactivity was found in the CRC cells compared to unchanged tissues from CRC patients; however, it was not associated with a poor prognosis. Benya et al. showed that GalR1 mRNA expression was regulated in human colonic epithelial T84 cells by the transcription nuclear factor-κB (NF-κB) and binding of galanin resulted in the secretion of Cl^−^ ions [14]. Other authors indicated that the GalR1 mRNA may be upregulated as a consequence of inflammatory conditions, probably as a result of NF-κB activation [24]. 

The assessment of the methylation status of galanin and its receptors in CRC could provide an additional prognostic marker since hypermethylation of galanin and GalR1 and GalR2 genes were found to correlate with larger tumor size and disease recurrence in HNSCC patients [25,26]. In contrast, activation of GalR1 induces cell-cycle arrest and suppresses the proliferation of HNSCC cell lines [25,26] and SH-SY5Y neuroblastoma cell line [27].

In contrast to increased GalR1 and GalR3 immunoexpression, in our study, GalR2 protein expression was at the same level in CRC cells and unchanged tissue. Our data are similar to investigations on SH-SY5Y neuroblastoma cell lines [27] and on glioma and pituitary adenoma [28]. Falkenstetter et al. reported a lack of GalR2 protein immunoreactivity in contrast to high expression of GalR1 and GalR3 proteins in examined tumors and lower level of GalR2 mRNA than GalR1 and GalR3 mRNA [28]. However, in the SH-SY5Y neuroblastoma cells transfected with GalR2, galanin strongly decreased the cell viability resulting from the induction of apoptosis [27,29]. 

Galanin and GalR2/GalR3 agonists were shown to inhibit the growth of PC12 pheochromocytoma cells stably transfected with GalR2 by inducing their apoptosis [30]. Moreover, the transfection of GalR2 into HNSCC cells induced cell cycle arrest and caspase-3–dependent apoptosis and made them susceptible to galanin-induced growth inhibition and apoptosis [31]. However, the overexpression of GalR2 demonstrated that GalR2 stimulated angiogenesis in HNSCC animal model acting via p38-MAPK-mediated secretion of proangiogenic cytokines, VEGF, and interleukin-6 (IL-6) [32,33]. These data clearly show that the expression of GalR2 and its role in cancer progression depends on the type and origin of cancer.

Galanin receptors are responsible for the transduction of the galanin signal, however, each of the three types of GalRs is coupled to a variety of signal transduction pathways [11]. In our study, the GalR1 and GalR3 immunoreactivities were stronger in CRC cells compared to epithelial cells of the unchanged mucosa, but only GalR3 immunoreactivity was associated with the survival of CRC patients.

Based on data submitted to GDC Data Portal (TCGA-COAD project), we found that transcriptional analyses of human colorectal cancer revealed that the gene expressions of GalR1 and GalR2 were higher in unchanged tissue (*n* = 7) than in CRC cells (*n* = 129, *p* < 0.05). In contrast, the level of GalR3 mRNA was similar in CRC cells compared to unchanged epithelial cells (*p* > 0.05). Of note, for the TCGA-COAD database, the whole samples were homogenized (tumor and whole wall of the unchanged large intestine with the mucosa, submucosa, muscularis externa, and adventitia) clearly without distinction between the localization/distribution of colon wall layers. Therefore, our present findings concerning the immunoexpression of GalR proteins may not coincide with the results of gene expression studies. 

On the other side, this discrepancy could be attributed to transcriptional and post-transcriptional mechanisms (RNA processing and stability, differences in mRNA and protein stability, feedback that suppresses mRNA expression through high protein concentrations, and attenuates post-transcriptional processes through high levels of gene expression) [34]. Moreover, the correlations between gene and protein abundances in mammals usually do not exceed 40% [35]. 

The immunohistochemical technique seems to be a relevant method for the assessment of the prognostic significance of the analysis of many pathological tumor samples paired with the neighboring unchanged tissue. We observed the high immunoexpression of GalRs in immune cells infiltrated CRC cells and in adjacent tissue. Galanin is a potent modulator of cytokine and chemokine expression in human macrophages [36]. Tumor-associated macrophages represent one of the immune populations most frequently infiltrated in colorectal tumors [37]. The distribution of GalRs in CRC and in immune cells in proximity to CRC cells may be a key to understanding the special status of GalR3. The mechanisms of action of galanin via GalRs in CRC and infiltrating immune cells have to be explored in further studies.

## 4. Materials and Methods

### 4.1. Patients

A total of 55 CRC patients with histologically confirmed CRC, 31 men, and 24 women, aged 67 ± 10.95 years (mean ± standard deviation, range 32–97 years) operated on at the Warmia and Mazury Oncological Center (Olsztyn, Poland) between 2012 and 2016 were included in the present study. Resected colorectal tissues were collected postoperatively from the colon wall of CRC patients. None of the CRC patients suffered from inflammatory bowel disease (IBD) or other gastrointestinal diseases and no patient reported a family history of malignancy. 

None of the patients had suffered from a second neoplastic disease or other serious diseases. Patients that had undergone neoadjuvant radiotherapy or chemotherapy were excluded from the study. The clinical characteristics and overall survival (OS) data of the patients were collected during the study. The tumor stage was characterized according to the TNM system. The study was approved by the Bioethical Commission of the University of Warmia and Mazury (Olsztyn, Poland, approval no. 43/2011), and written consent was obtained from all participants.

### 4.2. Collection of Tumor and Colon Wall Samples

Immediately after resection of the part of the large intestine, full-thickness intestinal wall samples (size ~5–6 mm) were collected: one sample was obtained from the colon wall directly from the tumor and the second sample (control) was obtained from the proximal part of the intestine at a distance of min. ≥5 cm from the tumor. Immediately after dissection, the sections of the colon wall were fixed in 4% buffered paraformaldehyde for 48 h at room temperature (RT), dehydrated in increasing ethanol series, cleared with xylene, embedded in paraffin and cut into 5 µm-thick sections.

### 4.3. Immunohistochemistry and Evaluation of Immunoreactivity

Immunohistochemical analysis was performed as described previously by Kieżun et al. with modifications [38]. The sections were subjected to an antigen retrieval procedure by microwaving for 12 min in Retrieval Solution Buffer, pH 6.0 (Leica Microsystems, Wetzlar, Germany), and then endogenous peroxidase activity was blocked by incubation with 3% H_2_O_2_ in methanol for 10 min. Next, the unspecific binding sites were blocked with 2.5% normal horse serum (Vector Laboratories, Burlingame, CA, USA) for 30 min. 

The sections were incubated overnight at 4 °C with rabbit polyclonal anti-human antibodies against GalR1 (Cat. No. GTX108207, GeneTex, Irvine, CA, USA), GalR2 (Cat. No. GTX100382, GeneTex, Irvine, CA, USA) and GalR3 (Cat. No. GTX10816, GeneTex, Irvine, CA, USA), all diluted 1:400 in phosphate-buffered saline (PBS). On the next day, sections were incubated with secondary antibodies (ImmPRESS Universal reagent Anti-Mouse/Rabbit Ig, Vector Laboratories, Burlingame, CA, USA) for 30 min. 

Schrődl et al. checked that there were no cross-reactions between the GalR1 and GalR3 antibodies that were also used in our study [39]. To visualize the immunoreaction, the sections were immersed in DAB (Dako, Carpinteria, CA, USA), then counterstained with hematoxylin (Sigma-Aldrich, St. Louis, MO, USA), dehydrated in ethanol, cleared in xylene and mounted. The immunostained sections were photographed using an Olympus BX-53 microscope and XC-50 camera (Olympus Corp., Tokyo, Japan). The specificity of immunohistochemical staining was checked by omitting the primary antibody and by replacing it with the same dilution of rabbit serum. 

The immunoreactivity of GalRs was assessed by two different pathologists, who was blinded to the patient clinical data, in the cytoplasm of CRC cells and epithelial cells of the unchanged colon mucosa (control sections), using the scale based on the reaction intensity (0, no reaction; 10, ≤10%; 30, 11–30%; 60, 31–60%; 80, 61–80%; and 100, >80%) as described previously [40]. In the cases of different assessments, the third pathologist checked the sections.

The immunofluorescent staining was performed as described by Kisielewska et al. [41]. Briefly, the tumor tissue and unchanged tissue (*n* = 5) were fixed, washed and cryoprotected for 1–2 days in graded solutions (19% and 30%) of sucrose (Sigma-Aldrich, St. Louis, MO, USA) at 4 °C. The tissues were frozen and cut into 5 μm thick cryostat coronal sections and stored at −80 °C. The sections were air-dried, washed and incubated for 1 h with blocking buffer (0.1 M PBS, 10% normal donkey serum, 0.01% bovine serum albumin, 1% Tween, 0.05% thimerosal and 0.01% NaN3). 

Then, the sections were incubated overnight with rabbit polyclonal antibodies against GalR1 (Cat. No. GTX108207, GeneTex, USA), GalR2 (Cat. No. GTX100382, GeneTex, USA) and GalR3 (Cat. No. GTX10816, GeneTex, USA), all diluted 1:400 in phosphate-buffered saline (PBS) and then for 1 h with the Alexa Fluor 555 donkey antirabbit antibodies (1:1000, A-31572, Molecular Probes, Eugene, OR, USA). To visualize the nuclei, the sections were mounted in Fluoroshield with DAPI (Merck, Kenilworth, NJ, USA). The sections were photographed using an Olympus BX-53 microscope with an Olympus DP23 camera and analyzed by CellSense v3.2 software (Olympus Corp., Tokyo, Japan). The specificity of immunofluorescent staining was checked by omitting the primary antibody.

Protein isolation and Western Blot analysis were performed as we described earlier [42]. Briefly, paired samples of tumor tissue and unchanged tissue derived from five CRC patients were homogenized, centrifuged and total protein content was determined. Samples were aliquoted and stored at −80 °C until further analyses. To determine the level of GalR1-3 proteins in tissue lysates, the SDS-PAGE followed by western blotting assays were performed. Proteins were transferred onto PVDF membrane (western blotting membrane, Roche, Mannheim, Germany). 

Blots were blocked and overnight incubated at 4 °C with respective primary antibody (rabbit polyclonal anti-human antibodies against GalR1, Cat. No. GTX108207, GeneTex, USA; GalR2, Cat. No. GTX100382, GeneTex, USA and GalR3, Cat. No. GTX10816, GeneTex, USA, all diluted 1:800 in TBS-T and polyclonal antibodies anti-actin, ACTB; 1:100; #A2066, Sigma-Aldrich) were used. ACTB level was used as the internal protein loading control. 

Then, the membranes were treated with the specific HPR-conjugated goat anti-rabbit IgG secondary antibodies (1:40,000; #A0545, Sigma-Aldrich) for 90 min at room temperature (RT), developed with an enhanced chemiluminescence (SuperSignal West Pico Chemiluminescent Substrate, Thermo Fisher Scientific, Waltham, MA, USA) and visualized with G:BOX iChemi XR imaging system (Syngene, Cambridge, UK). For the negative control, a primary antibody was omitted and substituted with phosphate-buffered saline (PBS). A molecular weight standard (Spectra Multicolor Broad Range Protein Ladder, Thermo Fisher Scientific, Waltham, MA, USA) was included into each blotting experiment to confirm the molecular weights of the detected bands.

### 4.4. Statistical Analyses

All statistical analyses regarding the immunoexpression of GalR proteins by immunohistochemistry were performed using Statistica software version 13.1 (StatSoft, Inc., Tulsa, OK, USA). The results were expressed as the mean ± standard error of the mean (SEM). *p* < 0.05 was considered to indicate a statistically significant difference. The differences between the immunoreactivity of GalRs in CRC cells and epithelial cells of non-cancerous colon mucosa were detected by a Wilcoxon matched-pairs test. The Mann–Whitney U test, Fisher’s exact test and Spearman’s rank correlation were used to assess associations between clinical-pathological data and the immunoexpression of GalRs in colorectal cancer cells compared to the epithelial and stromal cells of the unchanged mucosa of the same CRC patients. 

Survival curves were plotted according to the Kaplan–Meier method. The significance of differences in overall survival (OS) between groups of patients was evaluated by a log-rank test. The uni- and multivariate survival associations were analyzed using the Cox proportional hazards regression model. Based on the relative GalR immunoreactivity, the specimens were divided into two groups regarded as ‘down-regulated’ (relative GalR immunoreactivity < 1) and ‘no change or up-regulated’ (relative GalR immunoreactivity ≥ 1).

### 4.5. Expression Profiling of GalR Family Genes in Colorectal Patients

The gene expression profiles of GalRs were obtained from the TCGA-COAD project deposited in GDC Data Portal (https://portal.gdc.cancer.gov/, accessed on 15 November 2021). Normalized expression values were retrieved and compiled using curated CRC Data (https://bioconductor.org/packages/release/data/experiment/html/curatedCRCData.html, accessed on 15 November 2021) R Bioconductor packages. The patients with defined sample types (tumor and adjacent normal tissue) were selected from the TCGA-COAD_eset dataset and clustered according to their expression profiles. To visualize the data, the heatmap2 function implemented in the gplots R package was applied.

## Figures and Tables

**Table 1:** Associations between demographic and clinical-pathological features of colorectal cancer (CRC) patients and relative protein expression determined by immunohistochemistry.

Qualitative Parameters	Number of Cases n (%)	GalR1-Ir Tumors vs. No Change Epithelial Tissues			GalR2-Ir Tumors vs. No Change Epithelial Tissues			Number of Cases n (%)	GalR3-Ir Tumors vs. No Change Epithelial Tissues		
		<1 n (%)	≥1 n (%)	*p*-Value	<1 n (%)	≥1 n (%)	*p*-Value		<1 n (%)	≥1 n (%)	*p*-Value
Total	55 (100)	12 (21.8)	43 (78.2)		15 (27.3)	40 (72.7)		55 (100)	8 (14.5)	47 (85.5)	
Men	31 (56)	9 (29.0)	22 (71.0)	0.1943	10 (32.3)	21 (67.7)	0.3800	31 (56)	5 (16.1)	20 (83.9)	1.0000
Women	24 (44)	3 (12.5)	21 (87.5)		5 (20.8)	19 (79.2)		24 (44)	3 (12.5)	21 (87.5)	
Age											
≤median (66 y.o.)	28 (51)	6 (21.4)	22 (78.6)	1.0000	7 (25.0)	21 (75.0)	0.7681	28 (51)	4 (14.3)	24 (85.7)	1.0000
>median (66 y.o.)	27 (49)	6 (22.2)	21 (77.8)		8 (29.6)	19 (70.4)		27 (49)	4 (14.8)	23 (85.2)	
Localization											
cecum, right colon	21 (38)	6 (28.6)	15 (71.4)	0.1439	5 (23.8)	16 (76.2)	0.1661	21 (38)	2 (9.5)	19 (90.5)	0.7072
transverse left colon, sigmoid	23 (42)	6 (26.1)	17 (73.9)		9 (39.1)	14 (60.9)		23 (42)	(17.4)	19 (82.6)	
rectum	11 (20)	0 (0.0)	11 (100.0)		1 (9.1)	10 (90.9)		11 (20)	2	9 (81.8)	
T status											
T1+T2	44 (80)	11 (25.0)	33 (75.0)	0.4223	9 (20.5)	35 (79.5)	0.0523	44 (80)	4 (9.1)	40 (90.9)	**0.0422**
T3	11 (20)	1 (9.1)	10 (90.9)		6 (54.5)	5 (45.5)		11 (20)	4 (36.4)	7 (63.6)	
N status											
N0	31 (56)	9 (29.0)	22 (71.0)	0.1943	6 (19.4)	5 (10)	0.2216	31 (56)	1 (3.2)	30 (96.8)	**0.0159**
N1+N2	24 (44)	3 (12.5)	21 (87.5)		9 (37.5)	1 (6)		24 (44)	7 (29.2)	17 (70.8)	
Distant metastases											
M0	47 (85)	11 (23.4)	36 (76.6)	0.6695	9 (19.1)	6 (11)	**0.0034**	47 (85)	3 (6.4)	44 (93.6)	**0.0008**
M1	8 (15)	1 (12.5)	7 (87.5)		6 (75.0)	0 (0)		8 (15)	5 (62.5)	3 (37.5)	

Ir-immunoreactivity; significant *p*-values (<0.05) are given in bold.

**Table 2:** Univariate and multivariate Cox regression analysis of the overall survival rates associated with different prognostic variables in patients with colorectal cancer (CRC).

Parameter	Univariate Cox Regression			Multivariate Cox Regression		
	HR	95% CI	*p*-Value	HR	95% CI	*p*-Value
relative protein GalR1-Ir (down-regulated vs. no change/up-regulated)	0.47	0.06–3.72	0.4731			
relative protein GalR2-Ir (down-regulated vs. no change/up-regulated)	1.83	0.52–6.51	0.3475			
relative protein GalR3-Ir (down-regulated vs. no change/up-regulated)	**5.57**	**1.56–19.92**	**0.0082**	0.15	0.02–1.14	0.0663
Gender (women vs. men)	1.29	0.36–4.60	0.6931			
Age (years)	0.91	0.25–3.22	0.8775			
Localization (rectum vs. cecum, right colon) (rectum vs. transverse left colon, sigmoid)	0.63 0.15	0.17–2.34 0.02–1.31	0.4900 0.0600			
Depth of invasion (T3 vs. T1+T2)	3.44	0.97–12.22	0.0600			
Lymph node metastasis (N1+N2 vs. N0)	**5.97**	**1.27–28.12**	**0.0200**	2.06	0.29-14.62	0.4704
Distant metastases (M1 vs. M0)	**17.39**	**4.78–63.32**	**0.00002**	**56.50**	**1.27–12.6**	**0.0006**

Median follow-up time: 62.5 months; Significant *p*-valus (*p* < 0.05); HR: hazard ratio; CI: confidence interval; Ir-immunoreactivity; significant *p*-values (<0.05) are given in bold. Ir-immunoreactivity.

## Data Availability

Data collected from the database (TCGA-COAD project deposited in GDC Data Portal, https://portal.gdc.cancer.gov/, accessed on 15 November 2021).
